# Integrated Human Skin Bacteria Genome Catalog Reveals Extensive Unexplored Habitat‐Specific Microbiome Diversity and Function

**DOI:** 10.1002/advs.202300050

**Published:** 2023-08-07

**Authors:** Zhiming Li, Yanmei Ju, Jingjing Xia, Zhe Zhang, Hefu Zhen, Xin Tong, Yuzhe Sun, Haorong Lu, Yang Zong, Peishan Chen, Kaiye Cai, Zhen Wang, Huanming Yang, Jiucun Wang, Jian Wang, Yong Hou, Xin Jin, Tao Zhang, Wenwei Zhang, Xun Xu, Liang Xiao, Ruijin Guo, Chao Nie

**Affiliations:** ^1^ BGI Research Shenzhen 518083 China; ^2^ China National GeneBank BGI Research Shenzhen 518120 China; ^3^ Shenzhen Key Laboratory of Neurogenomics BGI Research Shenzhen 518083 China; ^4^ State Key Laboratory of Genetic Engineering, Collaborative Innovation Center for Genetics and Development, and Human Phenome Institute Fudan University Shanghai 200438 China; ^5^ College of Life Sciences University of Chinese Academy of Sciences Beijing 100049 China; ^6^ Greater Bay Area Institute of Precision Medicine (Guangzhou) School of Life Sciences, Fudan University Guangzhou 511458 China; ^7^ James D. Watson Institute of Genome Sciences Hangzhou 310058 China; ^8^ The Cancer Hospital of the University of Chinese Academy of Sciences (Zhejiang Cancer Hospital), Institute of Basic Medicine and Cancer (IBMC) Chinese Academy of Sciences Hangzhou 310022 China; ^9^ Research Unit of Dissecting the Population Genetics and Developing New Technologies for Treatment and Prevention of Skin Phenotypes and Dermatological Diseases (2019RU058) Chinese Academy of Medical Sciences Shanghai 200438 China; ^10^ Shenzhen Key Laboratory of Human commensal microorganisms and Health Research BGI Research Shenzhen 518083 China; ^11^ BGI Research Wuhan 430074 China; ^12^ Shenzhen Engineering Laboratory of Detection and Intervention of human intestinal microbiome BGI Research Shenzhen 518083 China; ^13^ Qingdao‐Europe Advanced Institute for Life Sciences BGI Research Qingdao 266555 China

**Keywords:** bacterial genomes, mutation, secondary metabolites, single nucleotide, skin microbiome

## Abstract

The skin is the largest organ in the human body. Various skin environments on its surface constitutes a complex ecosystem. One of the characteristics of the skin micro‐ecosystem is low biomass, which greatly limits a comprehensive identification of the microbial species through sequencing. In this study, deep‐shotgun sequencing (average 21.5 Gigabyte (Gb)) from 450 facial samples and publicly available skin metagenomic datasets of 2069 samples to assemble a Unified Human Skin Genome (UHSG) catalog is integrated. The UHSG encompasses 813 prokaryotic species derived from 5779 metagenome‐assembled genomes, among which 470 are novel species covering 20 phyla with 1385 novel assembled genomes. Based on the UHSG, the core functions of the skin microbiome are described and the differences in amino acid metabolism, carbohydrate metabolism, and drug resistance functions among different phyla are identified. Furthermore, analysis of secondary metabolites of the near‐complete genomes further find 1220 putative novel secondary metabolites, several of which are found in previously unknown genomes. Single nucleotide variant (SNV) reveals a possible skin protection mechanism: the negative selection process of the skin environment to conditional pathogens. UHSG offers a convenient reference database that will facilitate a more in‐depth understanding of the role of skin microorganisms in the skin.

## Graphical abstract

Researchers recover 2111 high‐quality genomes and 3668 medium‐quality from 2519 human skin metagenomes, clustering yields 813 prokaryotic species including 470 novel species. Genomic analyses reveal core functions among taxa and different antibiotic resistances. The study identifies 1525 putative secondary metabolite biosynthesis gene clusters. Single‐nucleotide variation analyses show negative selection on potential pathogens. The genome catalog facilitates deeper skin microbiome studies.

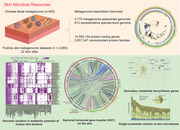

## 1 Introduction

In humans, skin health and diseases are related to the skin microbiome.^[^ ^1^ ^]^ Metagenomic analysis can deduce species classification and potential function of the complex microbiome to further understand its roles in skin health and disease.^[^ ^1^, ^2^ ^]^ However, the lack of sufficient reference data on microbial diversity hinders metagenomic data analysis and the comprehension of the potential function of microbial species. Culture research on skin microorganisms has constantly revealed fresh insight into the impact of skin community biology on human skin health.^[^ ^3^ ^]^ Although some unknown skin microorganisms may evade current culture methods for various reasons (such as lack of nutrients in the growth medium or low abundance of skin microorganisms), they may play an important biological role that has yet to be elucidated. Therefore, obtaining skin microbial genomes and establishing a comprehensive catalog is vital for novel discovery.

Several studies have demonstrated that culture‐independent and reference‐free methods are successful strategies for novel species discovery and characterization.^[^ ^4^ ^]^ Metagenomic analysis can involve binning de novo assembled contigs into hypothetical genomes, known as metagenomic assembly genome (MAG). Some studies have applied this method to reconstruct various MAGs,^[^ ^4^, ^5^ ^]^ one of which restored thousands of genomes of skin microorganisms in North American samples^[^ ^5a^ ^]^ and provided an exhaustive landscape of the diversity of the North American skin microbiome.

Here the Unified Human Skin Genome (UHSG) was generated from 2519 human skin metagenome samples. The UHSG includes 2111 high‐quality (HQ) MAGs and 3668 medium‐quality (MQ) MAGs. Next, 1385 novel assembled genomes were identified and studied for their unique potential functions. A further genomic clustering procedure resulted in 813 inferred prokaryotic species that included 549 near‐complete prokaryotic species (NCPS). In addition, the horizontal transfer of genes between species, the potential secondary metabolite biosynthesis, and single‐nucleotide variants was assessed based on the NCPS. These provided us with new insights into the species and functions of skin microorganisms and their crucial roles in the human skin.

## 2 Result

### 2.1 Large‐Scale Uncultured and Unassembled Bacterial Species Discovered in the Skin

To achieve exhaustive characterization of the human dermal microbiota, we performed deep shotgun sequencing of 450 healthy facial samples from the 4D‐SZ cohort (totaling 9.7 Terabyte (Tb) of high‐quality data, an average of 21.5 Gb of data) and retrieved 2069 human skin metagenomic datasets (totaling 6 Tb of high‐quality data, average 2.9 Gb data) from 4 different studies^[^ ^1^, ^2^, ^6^ ^]^ (Figure S1 and Table S1, Supporting Information). The available skin microbiome samples were mainly from the United States (*n* = 1099, 43.6%) or China (*n* = 1271, 50.5%); most samples were healthy samples, reflecting geographical and healthy sample favoritism in current human skin microbiome studies.

**Figure 1:**
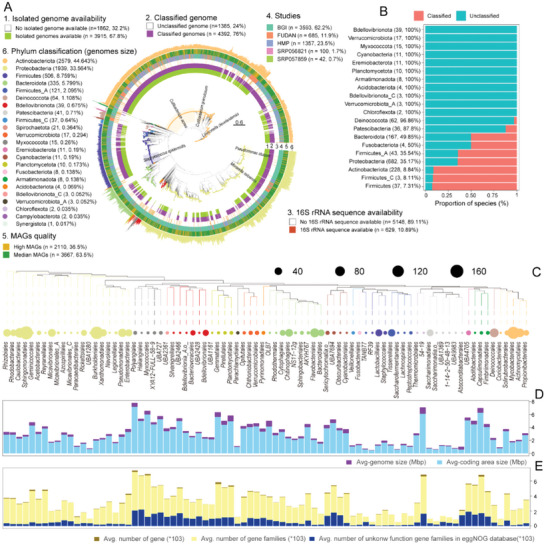
Phylogenetic diversity of the human skin microbiota genome catalog. A) Maximum‐likelihood phylogenetic tree of 5777 MAGs assembled in human skin. The outer circle of the clades describes the GTDB phylum annotation. MAGs are annotated by their isolated genome availability (1st layer), where they are classified as known species level (layer 2), 16S rRNA sequence availability (layer 3), from research project information (layer 4), MAGs quality control information (layer 5) and the bars represent the genome size (the outermost layer). B) The proportion of unclassified MAGs at the phylum level is displayed. The number of unclassified species at the phylum level and their proportion at the phylum level are represented in brackets. Diversity of composition and potential functional genes families of unclassified skin microbiota. C) The phylogenetic tree shows 82 (order levels) bacterial clades of the unclassified MAGs. The outer circle of the clades shows the number of MAGs for each clade. D) The average genome size and coding density of all species in each branch are displayed. E) The histogram shows the average number of genes, the average number of gene families, and the number of unknown functional genes of all species in each branch.

To obtain comprehensive, high‐quality reference genomes of the skin microbiome, we restored 27406 genomes using 2519 metagenome samples (Figure S1, Supporting Information). CheckM software was used to determine the genomes of contamination and completeness, whether high‐quality (HQ, completeness ≥ 90%, contamination ≤ 5%) or medium‐quality (MQ, completeness ≥ 50%, contamination ≤ 10%). Based on these metrics, 2111 HQ MAGs and 3668 MQ MAGs were obtained (UHSG, Figure S1 and Table S2, Supporting Information), which MAGs were obtained mainly from the samples collected in the 4D‐SZ cohort (Figure S1, Supporting Information, *n* = 3594, 62.2%). Afterward, each HQ and MQ MAG was classified using the Genome Taxonomy Database Toolkit.^[^ ^7^ ^]^ (GTDB‐Tk, **Figure** **1**A). However, 24% (*n* = 1385) of the MAGs could not be classified as a reported species, and 32.2% (*n* = 1862) of the MAGs were uncultured genomes of bacteria, establishing that the part of the UHSG is not currently represented in the reference databases (Figure 1A). Two of the 5779 MAGs were genomes of archaea, one of which could be classified as *Natronococcus amylolyticus*. The other could not be classified as an existing species, thus indicating the scarcity of archaea in the skin microbiome (Table S2, Supporting Information). UHSG adequately covered different taxonomic groups (Figure 1A), among the top 20 most abundant species in the skin (Figure S2, Supporting Information), using metagenomic data and microbial genome reconstruction methods, we obtained high‐quality genomes for *Cutibacterium acnes* (*n* = 1094), *Lawsonella clevelandensis* (*n* = 268), *Cutibacterium granulosum* (*n* = 255), *Staphylococcus epidermidis* (*n* = 193), *Pseudomonas stutzeri* (*n* = 190), and *Moraxella osloensis* (*n* = 156).

Next, we focused on “novel” genomes that were not assigned (95% average nucleotide identity (ANI)) to an existing species in GTDB (Figure 1B). The “novel” genomes were concentrated in Proteobacteria (682, 49.21%), Actinobacteriota (228, 16.45%), Bacteroidota (167, 12.05%), and Deinococcota (62, 4.47%), involving 82 orders and 139 families (Figure 1C and Figure S3, Supporting Information). Furthermore, the coding space (ranging from 57.1% to 95.0%), gene duplication rate (ranging from 0% to 5.9%), and “novel” potential function (ranging from 2.4% to 62.7%) of the genomes were highly diverse (Figure 1C, Figure S3 and Table S3, Supporting Information), supporting the important position of “novel” genomes in maintaining the functional diversity in the skin. In addition, novel species‐level branches within 11 phyla were found, which belong to phylum members of Bdellovibrionota, Verrucomicrobiota, Myxococcota, Cyanobacteria, Eremiobacterota, Planctomycetota, Armatimonadota, Acidobacteriota, Bdellovibrionota, Verrucomicrobiota, and Chloroflexota (Figure 1A,B). These branches were mainly assembled in high‐depth sequencing samples (Figure S4, Supporting Information), which suggested that high‐depth sequencing is beneficial to the genome assembly of unknown skin bacteria.

To determine the number of species that were included in UHSG, all HQ and MQ MAGs were clustered using at least 95% ANI and 30% alignment fraction thresholds.^[^ ^4^, ^8^ ^]^ Based on genome information (minimal contamination and maximum completeness), the best quality genomes from each cluster were chosen as representative species‐level genome bins (rSGBs). There were 813 rSGBs inferred from the clustering procedure, of which 549 were near‐complete prokaryotic species (NCPS, completeness ≥ 90%, contamination ≤ 5%) and 470 were “novel” species (Table S4, Supporting Information). The number of rSGBs was found to be far fewer than those of the reported gut microbiome^[^ ^4b^ ^]^ or oral microbiome,^[^ ^5b^ ^]^ discrepancy may be due to the number of rSGB has not reached a saturation point, indicating that more species remain to be discovered (Figure S5, Supporting Information). All skin metagenomic datasets were mapped to 813 rSGBs, confirming the representation of human skin microbial diversity in the UHSG. Using BWA‐MEM, a mean classification rate of 77.81% was obtained. In comparison to the HMP database, this represented a 35.5% improvement (Table S1, Supporting Information). Samples from China and the United States showed the most remarkable improvements in classification rate, highlighting the potential of the UHSG catalog for studying microbiome diversity among understudied populations.

### 2.2 Genomic Variation in Metabolic Potential of Human Skin Bacteria

**Figure 2:**
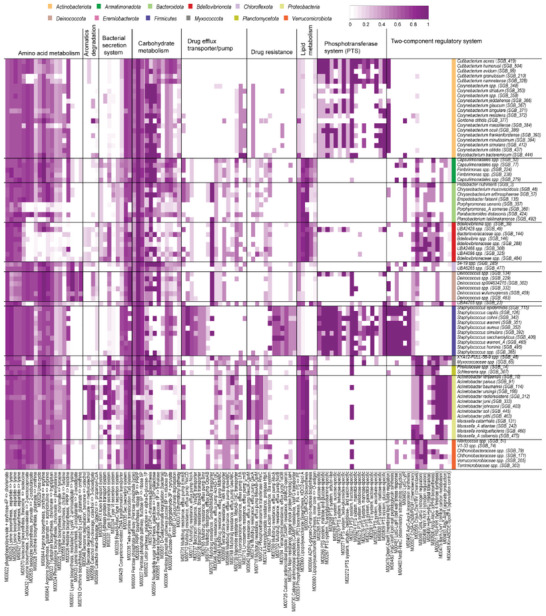
The potential differences in functions of skin bacteria at phylum level. The heatmap indicates the completeness rate of the KEGG metabolic module of NCPS in different phyla. The color depth shows the completeness ratio of KEGG metabolic modules in NCPS; dark purple, genes with coding all enzymes involved in the module were present; light purple, partially present; white, none.

Constructing a genomic catalog of skin bacteria is crucial to understanding the potential functions of skin bacteria.^[^ ^5^, ^6^ ^]^ The 5779 genomes encode a total of 14590154 protein‐coding genes (Table S2, Supporting Information), which can be clustered into 2657347 nonredundant protein families (NPFs). On average, 90.8% and 0.03% of genes showed sequence/domain homology in the eggNOG, and CARD, respectively (Table S2, Supporting Information). However, approximately 1351610 (50.86%) of the protein families in our NPF were not found in available iHSMGC.^[^ ^6a^ ^]^ In addition, principal coordinate analysis (PCoA) of the NPF profiles revealed a clear separation between the 17 bacterial phyla Actinobacteriota, Proteobacteria, Firmicutes, and Bacteroidota (Figure S6, Supporting Information). Unexpectedly, the *Cutibacterium granulosum* and other species taxonomy of *Cutibacterium* showed clear isolation, suggesting differences in their potential function of skin (**Figure** **2** and Figure S6, Supporting Information). Specifically, cofactor and vitamin biosynthesis, amino acid metabolism, phosphotransferase system, and lipid metabolism were significantly different among different species taxonomy of *Cutibacterium* (Table S5, Supporting Information).

The core functional pathways were reconstructed based on the completeness ratio of KEGG modules (Figure 2 and Figure S7, Supporting Information). All species had near‐complete central metabolism (i.e., glycolysis, gluconeogenesis, pentose phosphate pathway, and citric acid cycle), amino acid metabolism (i.e., lysine, proline, arginine, tryptophan, leucine, isoleucine, histidine and threonine biosynthesis), and nucleotide metabolism pathways (Figure S7, Supporting Information). The Actinobacteriota and Firmicutes phylum, especially *Cutibacterium* and *Staphylococcus* genus, displayed a more diverse phosphotransferase system (PTS) diversity than the other bacteria (Figure 2), suggesting their vital ability in the uptake of sugar and its derivatives. While Armatimonadota, Bacteroidota, Bdellovibrionota, Myxococcota, Planctomycetota, Proteobacteria (*Moraxella* and *Acinetobacter*), and Verrucomicrobiota showed richer functional diversity in lipid metabolism (Figure 2). In our previous study,^[^ ^6a^ ^]^ two skin cutotypes were found based on data‐driven categorization: the *C‐cutotype* comprised mainly *Cutibacterium*, and the *M‐cutotype* comprised mainly *Moraxella*. Here, through the functional analysis of the genome, the potential reason for the formation of two skin cutotype was that the two skin microbiota groups have different “nutrient requirements.”

More importantly, antibiotic resistance genes (ARGs) showed significant heterogeneity in different phyla (Figure 2). To evaluate the changes of ARGs between different phyla, NCPS genome sequences for ARGs were analyzed using the CARD database^[^ ^9^ ^]^ (v3.1.4) (Table S6, Supporting Information). The reservoir of ARGs in the skin was mainly from Proteobacteria and Firmicutes. The drug‐resistant reservoirs in the skin were found to be mainly in *Staphylococcus* in Firmicutes and *Pseudomonas*, *Acinetobacter, Klebsiella, Enterobacter himalayensis, Yokenella regensburgei*, and *Pluralibacter gergoviae* in Proteobacteria, which are multidrug‐resistant bacteria (Figure S8, Supporting Information). Most drug‐resistant bacteria were resistant to tetracycline antibiotics and fluoroquinolone antibiotics (Figure S8, Supporting Information), as these are widely used.^[^ ^10^ ^]^ Notably, the drug‐resistant bacteria of Proteobacteria in the skin were not resistant to fusidic acid, glycopeptide antibiotic, oxazolidinone antibiotic, para aminosalicylic acid, pleuromutilin antibiotic and streptogramin antibiotic. The drug‐resistant bacteria of Firmicutes were not resistant to benzalkonium chloride, bicyclomycin, carbapenem, cephamycin, elfamycin antibiotic, glycopeptide antibiotic, glycylcycline, isoniazid, monobactam, nitrofuran antibiotic, nitroimidazole antibiotic, and para aminosalicylic acid (Figure S8, Supporting Information). Our findings could guide antibiotics use in the treatment of skin diseases.

### 2.3 Bacterial Horizontal Gene Transfer (HGT) on the Skin

**Figure 3:**
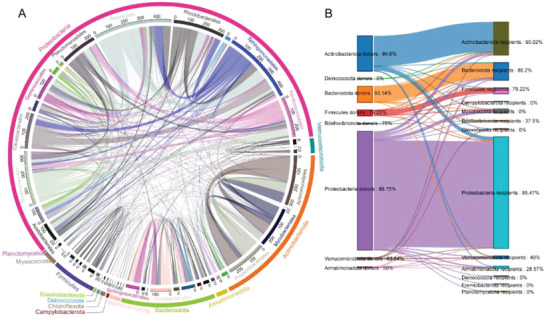
Horizontal gene transfer exists between species of bacteria. A) The innerring and band connecting donor and recipient are colored by the protein family of the gene being transferred, with the width of the band correlating to the number of HGTs. The inner ring is colored with microbial order. The outer ring is colored by microbial phylum. B) Sankey diagram depicts the horizontal transfer between phyla. Left: donor. Right: recipient. The percentage represents the horizontal transfer ratio between the same phyla.

In microbes, HGT is thought to drive microbial evolution and adaptation, which is related to the development of antibiotic resistance and virulence.^[^ ^11^ ^]^ A total of 2276 candidate HGT events (Table S7, Supporting Information) were identified in 422 NCPS using the MetaCHIP^[^ ^12^ ^]^ (based on best match and phylogenetic approaches). Out of these, the donors mainly included Proteobacteria (66%), Actinobacteriota (19.7%), Bacteroidota (9%), Firmicutes (3.4%), and Verrucomicrobiota (1%). The recipients mainly included Proteobacteria (62.1%), Actinobacteriota (18.5%), Bacteroidota (9.8%), Firmicutes (3.4%), Myxococcota (2.4%), Verrucomicrobiota (1.5%), and Armatimonadota (1.2%) (**Figure** **3**A). In particular, most of the transfers were noted inside the same phylum, and the species *Campylobacterota, Myxococcota, Chloroflexota, Eremiobacterota*, and *Planctomycetota w*ere only the recipients of the transferred gene (Figure 3B). In addition, a significant increase was noted in the number of HGT of species with relatively low abundance in the skin, such as *Bosea spp. (SGB_493), UBA1936 spp. (SGB_290), Aureimonas altamirensis (SGB_269), Chryseolinea spp. (SGB_153), Aquabacter spp. (SGB_209), Inquilinaceae spp. (SGB_201)*, and *Roseomonas mucosa (SGB_123)* (Figure S9, Supporting Information), thus suggesting their evolution and adaptation to the skin environment.

There were 238 horizontally transferred genes (10.5%) with a genetic divergence of less than 1%, indicating recent transfers (Figure S10A, Supporting Information). MetaCHIP also identified 2038 (89.5%) gene transfers with a genetic divergence higher than 1%, i.e., representing non‐recent transfers (Figure S10B, Supporting Information). Then, the COG system was used to annotate recently and non‐recently detected HGTs predicted by MetaCHIP software.^[^ ^12^ ^]^ The results showed that COG categories enriched with recent HGTs differ from those enriched with non‐recent HGTs. For example, COG categories C (energy production and conversion), Q (secondary metabolites biosynthesis, transport, and catabolism), and I (lipid transport and metabolism) were only enriched in the non‐recent HGTs, while categories K (transcription), L (replication, recombination, and repair), and U (intracellular trafficking, secretion, and vesicular transport) were enriched in recent HGTs (Figure S10B, Supporting Information). This varied from the previous results^[^ ^12^ ^]^ of HGT in soil and gut genomes. It shows that species in different growth environments have different needs for functional genes in the process of evolution.

HGT is a major factor contributing to the rapid spread of resistance.^[^ ^13^ ^]^ Six drug resistance genes (adeF, mexQ) were identified in 2276 candidate HGT events (Figure S11, Supporting Information). The multidrug resistance gene (mexQ) of *Pseudomonas aeruginosa*, considered a global health issue,^[^ ^14^ ^]^ was found to be transferred to *Serratia marcescens_1*, suggesting the need to pay attention to the transmission of multidrug resistance genes. In addition, the adeF gene with tetracycline and fluoroquinolone resistance^[^ ^15^ ^]^ may be widespread among skin bacteria, implying that the type of antibiotics used on the skin surface needs to be carefully considered.

### 2.4 Secondary Metabolite Biosynthesis Genes Are Widely Distributed among Skin Bacteria

**Figure 4:**
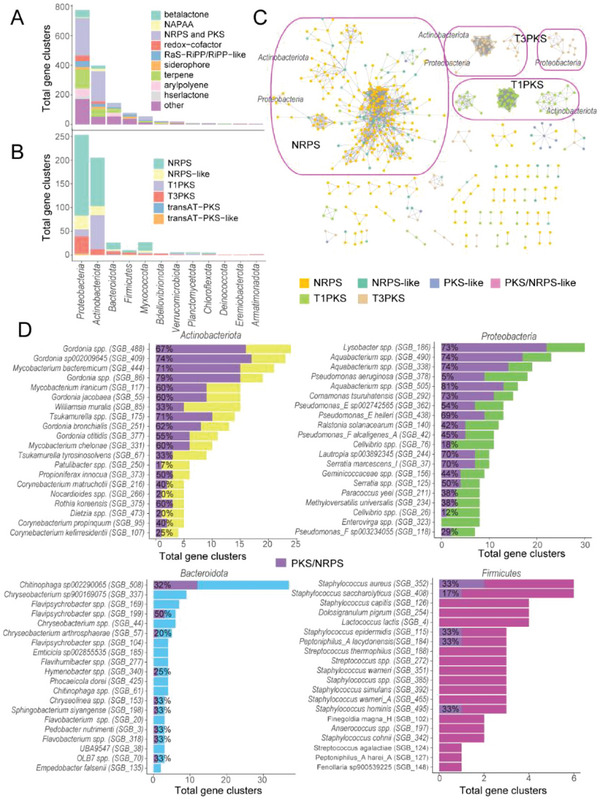
Diversity of potential biosynthetic gene clusters in skin bacteria. A) The number of biosynthetic gene clusters found on NCPS in each phylum, colored by putative product types as assigned by antiSMASH. B) The number of NRPS, PKS, NRPS‐like, and PKS‐like gene clusters found on NCPS of each phylum. C) Network of NRPS and PKS biosynthetic gene clusters, edges connect clusters that share genes. The line thickness increases with the increase of genetic similarity. Only the shared gene network of NRPS and PKS biosynthetic gene clusters predicted at one or two phyla levels is circled in purple. D) Biosynthetic gene clusters and NRPS/ PKS found on each NCPS. Purple: NRPS/PKS, other colors: biosynthetic gene clusters.

In skin ecosystems, microorganisms produce various secondary metabolites, such as antibiotics, antifungals, beta‐lactone, and siderophores that play a role in interacting with humans and the environment.^[^ ^16^ ^]^ While most known antibiotics were obtained through a few cultured microbial taxa,^[^ ^17^ ^]^ the potential secondary metabolites of most skin bacteria have hardly been investigated.^[^ ^16a^ ^]^ Here, 1525 biosynthetic gene clusters (BGCs) were identified at 474 NCPS using antiSMASH 6.0.0^[^ ^18^ ^]^ and DeepBGC^[^ ^19^ ^]^ (Table S8, Supporting Information). These gene clusters mainly included nonribosomal peptide synthetases (NRPSs) and/or polyketide synthases (PKSs), arylpolyene, betalactone, siderophore, terpene, hserlactone, non‐alpha poly‐amino acids (NAPAA), redox‐cofactor and post‐translationally modified peptides (RiPPs) (**Figure** **4**A). Only 20% of BGCs (305 of 1525) were found in the biosynthetic gene cluster (MIBiG) database (similarity ≥ 30%^[^ ^20^ ^]^) and reported in the previous studies (Table S8, Supporting Information). Therefore, our analysis greatly expanded the amount of potential secondary metabolite biosynthesis of skin bacteria.

Currently, the natural products of most known bacteria have been from the isolates of Actinobacteria and Proteobacteria, which represent bacteria that usually comprise the majority among skin microbial members.^[^ ^2^, ^6^ ^]^ In this study, BGCs were identified in 12 phyla of skin microorganisms, including Proteobacteria, Actinobacteria, Bacteroidota, Firmicutes, and Myxococcota (Figure 4B). The most abundant BGCs in Proteobacteria were *Lysobacter spp., Aquabacterium spp., Pseudomonas spp., Aquabacterium spp*., and *Comamonas tsuruhatensis;* the most abundant BGCs in Actinobacteria were *Gordonia spp*., *Mycobacterium spp*., *Tsukamurella spp*., *Williamsia muralis*, and *Patulibacter spp*.; the most abundant BGCs in Bacteroidota were *Chitinophaga spp*., *Chryseobacterium spp*., *Flavipsychrobacter spp*., *Emticicia sp002855535*, and *Hymenobacter spp*.; the most abundant BGCs in Firmicutes were *Streptococcus spp*., *Lactococcus spp*., *Dolosigranulum pigrum, Peptoniphilus_A spp*., and *Anaerococcus spp*. (Figure 4D).

Gene clusters encoding PKSs and NRPSs can produce various types of antibiotics, antifungals, siderophores, and immunosuppressants.^[^ ^21^ ^]^ Here, 639 NRPS, PKS (types I and III), and hybrid (NRPS‐PKS) gene clusters were identified on contigs from 239 NCPS (Figure 4B and Table S8, Supporting Information). Consistent with the above, these clusters mostly appeared in Proteobacteria, Actinobacteriota, Bacteroidota, Firmicutes, and Myxococcota (Figure 4B and Table S8, Supporting Information). The NRPS, PKS, and NRPS‐PKS gene clusters accounted for 32.81%, 51.64%, 12.82%, and 17.93% of the average BGCs in Proteobacteria, Actinobacteriota, Firmicutes, and Bacteroidota, respectively (Figure 4D).

Next, a cluster relational network was constructed based on the shared gene to compare the degrees of shared genes in biosynthetic clusters. This method revealed the diversity of potential secondary metabolites in the skin, and many diverse and sparsely connected BGCs in the skin microbiota were found (Figure 4C and Figure S12, Supporting Information). The relational network of clusters was mainly formed between species within Actinobacteriota, Proteobacteria, Firmicutes, and Bacteroidota (Figure 4C and Figure S12, Supporting Information). For example, A conserved type I PKS locus nearly ubiquitous in the Actinobacteriota formed three dense network clusters. At the same time, the conserved type III PKS locus was a dense network cluster in Proteobacteria (Figure 4C). Proteobacteria formed three aryl‐polyene, and four beta‐lactone, six terpene; Actinobacteria formed two beta‐lactone and three redox−cofactor conserved clusters; Bacteroidota formed conserved clusters of four aryl‐polyene and two terpenes (Figure S12, Supporting Information). The high conservatism of these BGCs in the taxa may indicate the presence of a novel group of metabolites.

### 2.5 Single‐Nucleotide Variants (SNVs) of Skin Microbiome

**Figure 5:**
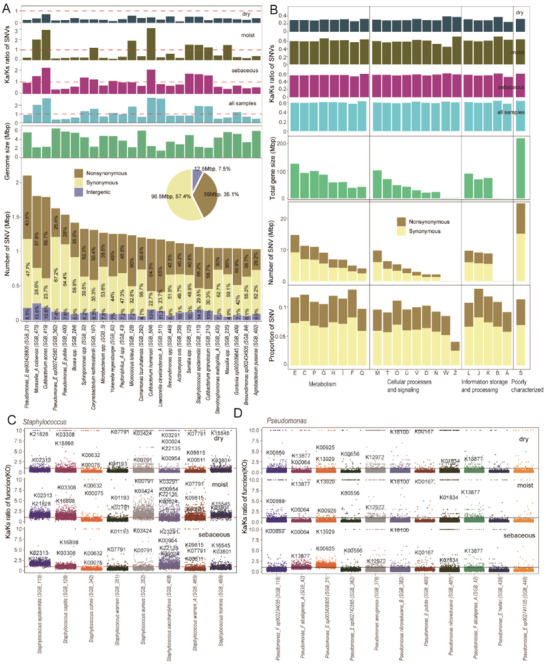
SNV density analysis of skin microbes. A) Histogram diagram depicts the distribution of the top 25 NCPS ranked by the number of SNVs. The color shows the number of SNVs in synonymous, non‐synonymous, and intergenic. The two histograms above show the genome size of the top 25 NCPS and Ka/Ks ratio in different skin environments. The pie chart shows the total number and ratio of SNVs in synonymous, non‐synonymous, and intergenic. B) The total gene size, SNVs number (synonymous and non‐synonymous), Ka/Ks ratio of function in different skin environments, and proportion of SNVs number to total gene size of different functional units is described through the categories of eggNOG. C) The Ka/Ks differences in potential functions between different species of *Staphylococcus* in different skin environments. D) The Ka/Ks differences in potential functions between different species of *Pseudomonas* in different skin environments.

To further explore the potential functions of skin bacteria, the single nucleotide variants of the NCPS were studied. The single‐nucleotide variants (SNVs) of the NCPS were quantified by inStrain.^[^ ^22^ ^]^ This yielded 168 076 666 SNVs from NCPS in 2519 samples, of which 57.4% were synonymous mutations (Ks), 35.1% were non‐synonymous mutations (Ka), and 7.5% occurred in the intergenic region (**Figure** **5**A). Most notably, the proportion of synonymous and non‐synonymous mutations (i.e., selective pressure) varied significantly between different species and skin environments. All bacteria exhibited negative selection in dry skin environments (Figure 5A). The selective pressure of the common skin bacteria *Cutibacterium acnes*, *Moraxella_A osloensis*, *Corynebacterium kefirresidentii*, *Cutibacterium humerusii*, *Staphylococcus epidermidis*, and *Cutibacterium granulosum* were positively selective in sebaceous and moist environments. While the selective pressure of pathogenic *Pseudomonas* (including *P. sp003428805, P. sp002742565*, and *P. putida*), *Bosea spp., Microbacterium spp*., and *Comamonas tsuruhatensis* were negatively selective in sebaceous and moist environments (Figure 5A). Additionally, we observed differential selection processes for certain bacteria across different skin environments. For instance, *Sphingomonas spp*. showed positive selection in sebaceous environments but negative selection in moist environments, *Massilia spp*. showed positive selection in moist environments but negative selection in sebaceous environments (Figure 5A). This finding suggests that conditional pathogenic bacteria are more inclined toward stabilizing selection, thus reducing the possibility of producing more toxic strains.

Next, SNVs were mapped to skin microbial gene function. It was found that SNVs appeared in almost all COGs, accounting for about 0.1 of the bacterial genome size, and the selection pressure of all COGs was negative selection, with lower negative selective pressure in dry environments compared to sebaceous and moist environments (Figure 5B). More importantly, the selective pressure of various functions of a single species was evaluated based on the genus level. Differences were noted in the selective pressure among different species of *Staphylococcus*, *Pseudomonas*, *Corynebacterium*, *Acinetobacter*, *Moraxella*, and *Cutibacterium* (Figure 5C,D and Figure S13, Supporting Information). These differences likely seem to be related to the conditional pathogenicity of bacteria, especially the negative selection pressure of functional genes of most *Pseudomonas* and *Staphylococcus aureus* concerning various diseases.^[^ ^23^ ^]^ Furthermore, they also display differences in selective pressure across different skin environments. The negative selective pressure observed in species of *Staphylococcus*, *Pseudomonas*, *Corynebacterium*, *Acinetobacter*, *Moraxella*, and *Cutibacterium* in dry environments (Figure 5C,D and Figure S13, Supporting Information) further explains the lower bacterial burden in dry environments of the skin compared to sebaceous and moist environments.^[^ ^24^ ^]^ Similarly, there are differences in selective pressure between sebaceous and moist environments. For instance, in moist environments, *Staphylococcus aureus*, *Staphylococcus hominis*, *Staphylococcus capitis*, *Acinetobacter radioresistens*, and *Moraxella_A atlantea* exhibit higher selective pressure compared to sebaceous environments. On the other hand, *Pseudomonas_E sp003428805*, *Corynebacterium macginleyi*, *Corynebacterium pseudodiphtheriticum*, and *Corynebacterium kroppenstedtii* shows lower selective pressure in sebaceous environments. In addition, many microbial functions showed positive selection pressure across the three skin environments, including proteins related to transport (K07791, K03291, K07791, K09815, K03308), transferases (K21828, K03801, K00791, K00954, K00611, K00556, K18100), kinase (K00859, K00925), and proteins related to carbohydrate metabolism (K22135, K00024, K01193, K13929, K13877, K00064, K00075, K12972) (Figure 5C,D). Our results emphasize that the interaction between skin bacteria and hosts may influence the direction of bacterial evolution.

## 3 Discussion

In this study, deep whole‐metagenomic shotgun sequencing of 450 Han skin samples was used in combination with 2069 skin samples to construct the UHSG comprising 2111 high‐quality genomes and 3668 medium‐quality genomes. Of the 813 prokaryote species in the set, 57.81% were “novel” species representatives, meaning that the majority of microbial diversity in the catalog still needs experimental characterization. During the preparation of this manuscript, a new collection of 622 skin prokaryotic species from culture and assembly were released,^[^ ^5a^ ^]^ of which 217 prokaryotic species were not in the UHSG set; these 217 species will be included in a future version. The UHSG is a resource that further expands and complements skin microbial studies, providing a comprehensive view of skin microbial diversity.

Skin microbial genomic functional analysis revealed the core functions of microbiota and the specific functions of different taxonomic levels. Actinobacteriota and Firmicutes, especially *Cutibacterium* and *Staphylococcus* genus, were found to exhibit a strong absorption capacity for sugar and its derivatives, and *Moraxella* and *Acinetobacter* in Proteobacteria showed richer lipid metabolism abilities, which is consistent with the driving bacteria in our previously reported skin microbial cutotypes.^[^ ^6a^ ^]^ More importantly, skin microorganisms displayed strong heterogeneity in potential drug resistance function. The drug‐resistant bacteria in the skin were more concentrated in Proteobacteria, especially *Pseudomonas aeruginosa*, which involves almost all kinds of antibiotics,^[^ ^25^ ^]^ and also showed strong resistance to various antibiotics in the skin. Accordingly, with *Pseudomonas aeruginosa* infections have become a genuine concern among hospital‐acquired conditions, especially in critically ill and immunocompromised patients.^[^ ^23a^ ^]^ Our findings revealed that *Pseudomonas aeruginosa* is not resistant to a few antibiotics, including elfamycin, fusidic acid, and glycopeptide. The use of these antibiotics can possibly reduce the mortality rates associated with *Pseudomonas aeruginosa* infections to a certain extent.

In recent years, it has become popular to mine secondary metabolites of microorganisms based on metagenomic, and numerous secondary metabolites have been identified from the gut, soil, and ocean microbiome.^[^ ^26^ ^]^ As the largest organ of the human body, the skin is in direct contact with the external environment. The microorganisms on the surface of the skin and the secondary metabolites they produce could play crucial roles in the human body. Several secondary metabolite synthesis genes were found in the genome of skin bacteria. As most of these secondary metabolites 80% could not be identified in the known database, it greatly expanded the secondary metabolite database. In addition, NRPSs and PKSs are essential natural products.^[^ ^27^ ^]^ because they have antimicrobial, antifungal, antiparasitic, anti‐tumor, and immunosuppressive properties gents with clinical value.^[^ ^28^ ^]^ It was found that the relationship network constructed by NRPS and PKS clusters can reach 60 independent subnets (Figure 4), each representing a bioactive molecule that may be developed and utilized. This finding can act as a reference for subsequent microbial drug development.

With the establishment of the genomic catalog of skin microorganisms, it is clear that more than half of the microbial species and functions have yet to be characterized. Through analysis, we characterized the function of bacteria in the skin, established the horizontal gene transfer between them, predicted the secondary metabolites, and characterized the SNVs of each bacterium and its adaptive evolution. Having such a comprehensive resource can help guide future research and prioritize targets for further experimental validation. By leveraging the information available in the resource, researchers can focus on specific microbial species, genetic pathways, or bioactive compounds that show potential for further investigation. In addition, according to the UHSG catalog, samples from community cohorts or specific clinical environments can reflect the abundance of skin bacteria. Through specific disease classification groups, targeted skin bacteria can be found to deepen the understanding of the role of skin bacteria in disease classification. With regards to the large amounts of uncultured bacteria in the skin, the UHSG catalog can significantly improve the research accuracy and resolution based on metagenome. Therefore, the genome presented in this study and its functional analysis will help elucidate the interactions between human health and skin microorganisms.

## 4 Experimental Section

### Ethics Statement

The study was approved by the BGI Review Board of Bioethics and Biosafety (BGI‐IRB19121). This study complied with all applicable institutional regulations concerning the ethical use of human information and samples. Each participant was required to provide their signed informed consent before enrolling in the program.

### Subject Recruitment and Sampling

In this study, 450 healthy volunteers were recruited from the 4D‐SZ cohort. Medication and medical treatment history were obtained from each person through questionnaires. Subjects with a history of skin diseases and antibiotic intake in the last six months were excluded. Each subject was requested not to use skincare or cosmetic products on the day before and the day of sampling. To maximize microbial skin load, samples were collected from the left and right cheeks of each subject. The room where the samples were collected was maintained at 20 °C and 50% humidity. The samples were collected and stored according to the method previously reported.^[^ ^6a^ ^]^

### DNA Extraction and Sample Sequencing

DNA extraction of the skin was performed according to the previously described MetaHIT protocol.^[^ ^6^, ^29^ ^]^ The DNA concentrations from skin samples were estimated by Qubit (Invitrogen). Due to the low bioburden typical of skin samples, the BGI NGS Platforms library was created using MGIEasy Universal DNA Library Prep Kit (BGI, Cat. No. 1000017571). The libraries were constructed using 0.5–10 ng of extracted DNA as input according to the manufacturer's recommended protocols. Libraries were then sequenced with 2*150 bp paired‐end reads on a DNBSEQ‐T1.

### Pre‐Processing and De‐Novo Assembly of Metagenomic Data

According to the previous description,^[^ ^6a^ ^]^ SOAPnuke^[^ ^30^ ^]^ was used for quality control, and bowtie2^[^ ^31^ ^]^ (v 2.3.5.1) was used to identify and delete human sequences similar to the human genome reference sequence (hg38). Metagenomic reads were assembled per sample using MEGAHIT^[^ ^32^ ^]^ (v1.1.3), which generated the initial assembly results based on different k‐mer sizes (*k* = 21, 33, 55, 77, 99). This resulted in 2.26*10^9^ different contigs for a total length of 1.88*10^11^ nt (Table S1, Supporting Information).

### Metagenome Binning and Quality Assessment

Using bowtie2^[^ ^31^ ^]^ (v2.3.5.1), high‐quality reads were mapped to contigs in each sample, and MetaBAT2^[^ ^33^ ^]^ (v.2.12.1) was used to bin each contig. Next, 27 406 bins with a total length of 4.74*10^10^ nt were generated through MetaBAT2. The “lineage_wf” workflow of CheckM^[^ ^34^ ^]^ (1.1.2) was used to calculate the completeness and contamination of each bin. High‐quality (HQ) and medium‐quality (MQ) genomes were classified according to the criteria of completeness ≥ 90%, contamination ≤ 5% and completeness ≥ 50%, contamination ≤ 10%, respectively. In total 2111 HQ MAGs and 3668 MQ MAGs were obtained. Barrnap^[^ ^35^ ^]^ (0.9) (default parameters) was used to search the 16S rRNA sequences in the MAG genomes. Only 10.89% (629) of the MAGs were recovered 16S rRNA (Figure 1A and Table S2, Supporting Information), consistent with the previously reported view that 16S rRNA was challenging to be recovered through metagenome assembly.^[^ ^4c^ ^]^

To evaluate the number of skin species, dRep (v2.2.4)^[^ ^36^ ^]^ was used to cluster 5779 MAGs (HQ and MQ). The parameters were as follows: “‐pa 0.9 ‐sa 0.95 ‐nc 0.30 ‐cm large.” A total of 813 inferred prokaryotic species were obtained from the clustering process, of which 549 were nearly complete prokaryotic species. The construction of species profiles was obtained by aligning high‐quality reads to 813 inferred prokaryotic species using bowtie2^[^ ^31^ ^]^ (v2.3.5.1). Subsequently, contigs of 813 inferred prokaryotic species coverage depths were calculated using “jgi_summarize_bam_contig_depths” with default parameters.^[^ ^33^ ^]^ Considering the different sequencing depths of different samples, normalized contig coverage depths were used to estimate contig relative abundance. Finally, the median of the relative abundance of contigs within the same species was utilized as a measure of the species relative abundance.

### Taxonomic Classification

GTDB‐Tk^[^ ^7a^ ^]^ (v1.3.1, GTDB database R05‐RS95) was used to classify workflow, and with “gtdbtk classify_wf ” was used to classify each MAG. The GTDB database release 95 covers nearly 200 000 genomes grouped into 31 910 species clusters. Protein sequence alignments generated by GTDB‐Tk were used to construct a maximum‐likelihood tree de novo. A phylogenetic tree was created using IQ‐TREE^[^ ^37^ ^]^ (v2.1.2) to illustrate the evolutionary relationships for the 5777 bacteria (two archaea were excluded). The Bayesian information criterion score of “ModelFinder” was used to automatically select the best‐fit model. Phylogenetic trees were constructed using LG+F+R9 models. Phylogenetic trees were visualized using the R package “ggtree.”

### Functional Characterization

The protein‐coding sequences (CDS) of 5779 genomes were predicted and annotated using prodigal^[^ ^38^ ^]^ (v2.6.3) and EggNOG^[^ ^39^ ^]^ (v5.0). For the pan‐genome analyses, firstly, orthologous gene clustering of the MAGs was performed using the MMseqs2 algorithm with options “–min‐seq‐id 0.5 ‐c 0.9” (similarity 50% and minimum coverage threshold of 90% of the length of the shortest sequence) at the protein level. Then, the protein level sequences of each MAG were compared with orthologous gene clustering using blastp (E‐value cutoff of 1*10^−6^ and an alignment length of at least 30%^[^ ^6a^ ^]^) function of the blast^[^ ^40^ ^]^ (v2.9.0+) to identify the common and specific sequences of 5779 MAGs.

### SNV Analyses

A total of 539 nearly complete prokaryotic species were used to generate a catalog of SNVs. The high‐quality reads were aligned to the nearly complete prokaryotic species by bowtie2^[^ ^31^ ^]^ Each sample generated a separate BAM file. The BAM file and nearly complete prokaryotic species were used as inputs and inStrain^[^ ^22^ ^]^ (v1.0.0) was used with default parameters (minimal coverage of a position of five reads, minimal frequency of an SNP of 0.05, and an FDR of 1*10^−6^) to identify synonymous and non‐synonymous SNVs. By the number of synonymous or non‐synonymous SNVs of species sequencing depth, the effect of sequencing depth between different samples was corrected for comparison between different groups.

### Horizontal Gene Transfer Analysis

MetaCHIP (v1.10.4)^[^ ^12^ ^]^ was used with default parameters to analyze HGTs of 539 nearly complete species levels. Candidate HGT genes used the results identified in the GTDB database^[^ ^7a^ ^]^ in the defined taxonomic groups, and the potential functions of candidate HGT genes were based on the results annotated by EggNOG.^[^ ^39^ ^]^.

### Biosynthetic Gene Cluster Analysis

With default parameters, antiSMASH^[^ ^18^ ^]^ (v6.0.0) and DeepBGC^[^ ^19^ ^]^ (v0.1.29) was used to process nearly complete prokaryotic species genomes. All potential secondary metabolite biosynthesis genes clusters are summarized in Table S8 (Supporting Information). The gene cluster network based on shared genes was established using a BLASTP^[^ ^40^ ^]^ search of predicted biosynthetic protein sequences. Shared proteins were defined as a protein alignment in which at least 50% of the query sequence was covered and the amino acid identity was >50%.

### Statistical Analysis

Principle coordination analysis (PCOA) was conducted based on the Bray–Curtis dissimilarity on the nonredundant protein families profile using the ade4 package^[^ ^41^ ^]^ to assess the differences in potential functional genes of MAGs at phylum, class, order, family, genus, and species levels. The effect sizes of bacterial phylogeny on potential functional were estimated based on permutational multivariate analysis of variance (PERMANOVA) via the R vegan package. The *p*‐values were generated by the Wilcoxon rank‐sum test unless specifically mentioned.

## Conflict of Interest

The authors declare no conflict of interest.

## Author Contributions

Z.L. and Y.J. were co‐first authors. C.N. and R.G. contributed equally to this work as co‐corresponding authors. C.N. and R.G. conceived and directed the project. X.X., W.Z., H.Y., J.W., Y.H., X.J. and L.X. initiated the overall health project. H.Z., Y.Z., P.C., Y.S., K.C., T.Z., and X.T. contributed to the organization of the cohort, the sample collection, and questionnaire collection. X.T. and Z.W. helped check the phenotypes. H.L., R.G., and Y.J. led the DNA extraction and sequencing. Z.L., R.G., and Y.J. led the bioinformatic analyses. Z.L performed the bioinformatic analyses and prepared figures and texts for the manuscript. Z.L. and R.G. interpreted the results and wrote the manuscript. J.X., Z.Z., J.W., T.Z., and Y.J. contributed to the revision of the manuscript. All authors read and approved the final manuscript.

## Supporting information

Supporting Information

Supplemental Table 1

## Data Availability

Metagenomics shotgun sequencing data and 5779 MAGs have been deposited into the CNGB Sequence Archive (CNSA; https://db.cngb.org/cnsa/) of China National GeneBank DataBase (CNGBdb) with accession number CNP0002131, reference number 42 and 43.
