# Supplementary material for: Integrated Human Skin Bacteria Genome Catalog Reveals Extensive Unexplored Habitat‐Specific Microbiome Diversity and Function
Source: Adv Sci (Weinh). 2023 Aug 7;10(28):2300050. doi: 10.1002/advs.202300050 (PMC10558695; doi:10.1002/advs.202300050)
Supplement: Supplementary file 1 — Supporting Information [file ADVS-10-2300050-s002.pdf]

ADVANCED SCIENCE
Open Access

Supporting Information

for *Adv. Sci.*, DOI 10.1002/advs.202300050

Integrated Human Skin Bacteria Genome Catalog Reveals Extensive Unexplored Habitat-Specific Microbiome Diversity and Function

Zhiming Li, Yanmei Ju, Jingjing Xia, Zhe Zhang, Hefu Zhen, Xin Tong, Yuzhe Sun, Haorong Lu, Yang Zong, Peishan Chen, Kaiye Cai, Zhen Wang, Huanming Yang, Jiucun Wang, Jian Wang, Yong Hou, Xin Jin, Tao Zhang, Wenwei Zhang, Xun Xu, Liang Xiao, Ruijin Guo and Chao Nie**

Supporting Information

Integrated Human Skin Bacteria Genome Catalog Reveals Extensive Unexplored Habitat-Specific Microbiome Diversity and Function

Zhiming Li, Yanmei Ju, Jingjing Xia, Zhe Zhang, Hefu Zhen, Xin Tong, Yuzhe Sun, Haorong Lu, Yang Zong, Peishan Chen, Kaiye Cai, Zhen Wang, Huanming Yang, Jiucun Wang, Jian Wang, Yong Hou, Xin Jin, Tao Zhang, Wenwei Zhang, Xun Xu, Liang Xiao, Ruijin Guo, Chao Nie**

This PDF file includes:

Supplementary Figure S1 to S11

Captions for Supplementary Materials Table S1-8

Other Supplementary Materials for this manuscript include the following:

Supplementary Materials Table S1-8 (Excel)

Supplementary Figures

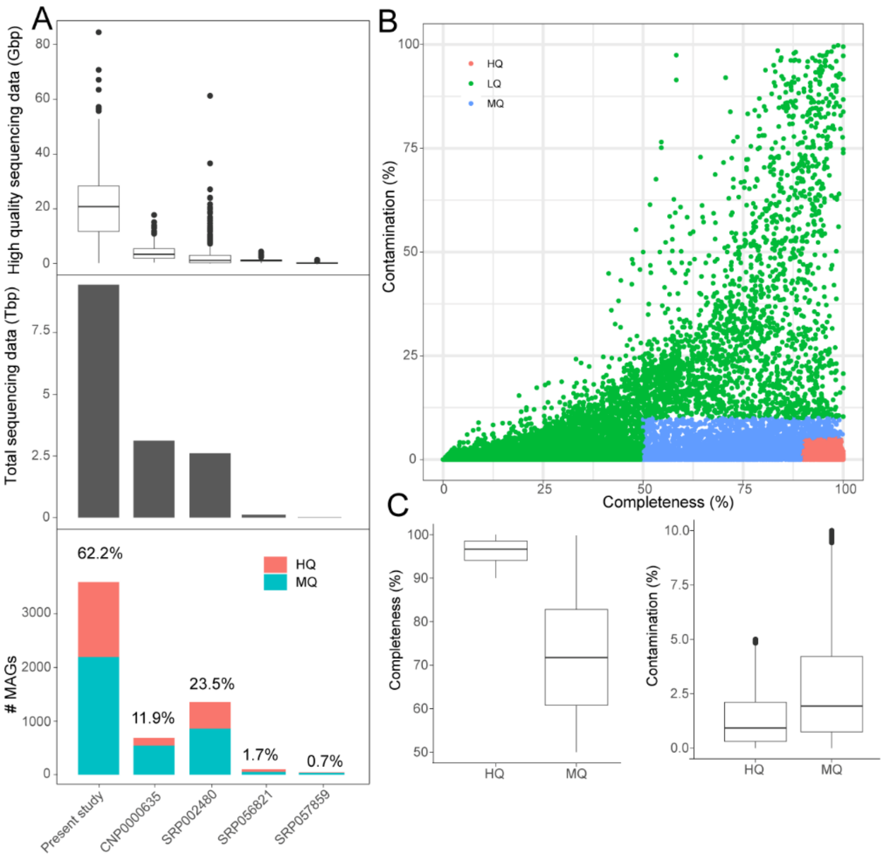

Figure S1. Characteristics of the reconstructed genomes in terms of quality.

(A) The histogram shows the high-quality sequencing data, the total sequencing data, and the number of MAGs assembled for each research project. **(B)** The quality of 27,406 bins was evaluated by CheckM. HQ: high-quality (completeness >= 90%, contamination <= 5%), MQ: medium-quality (completeness >= 50%, contamination <= 10%), LQ: low-quality (contamination > 10% or completeness < 50%) **(C)** A comparison between the completeness and contamination of reconstructed high-quality and medium-quality genomes.

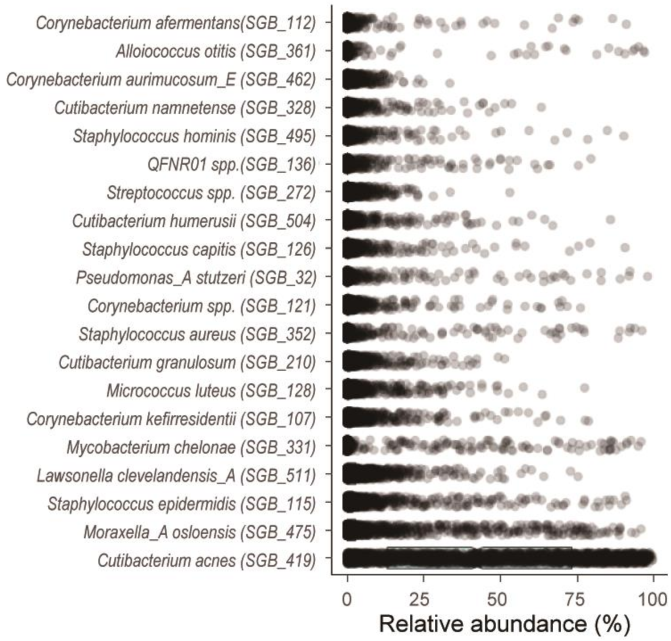

Figure S2. Top 20 most relative abundant rSGBs in all skin samples.

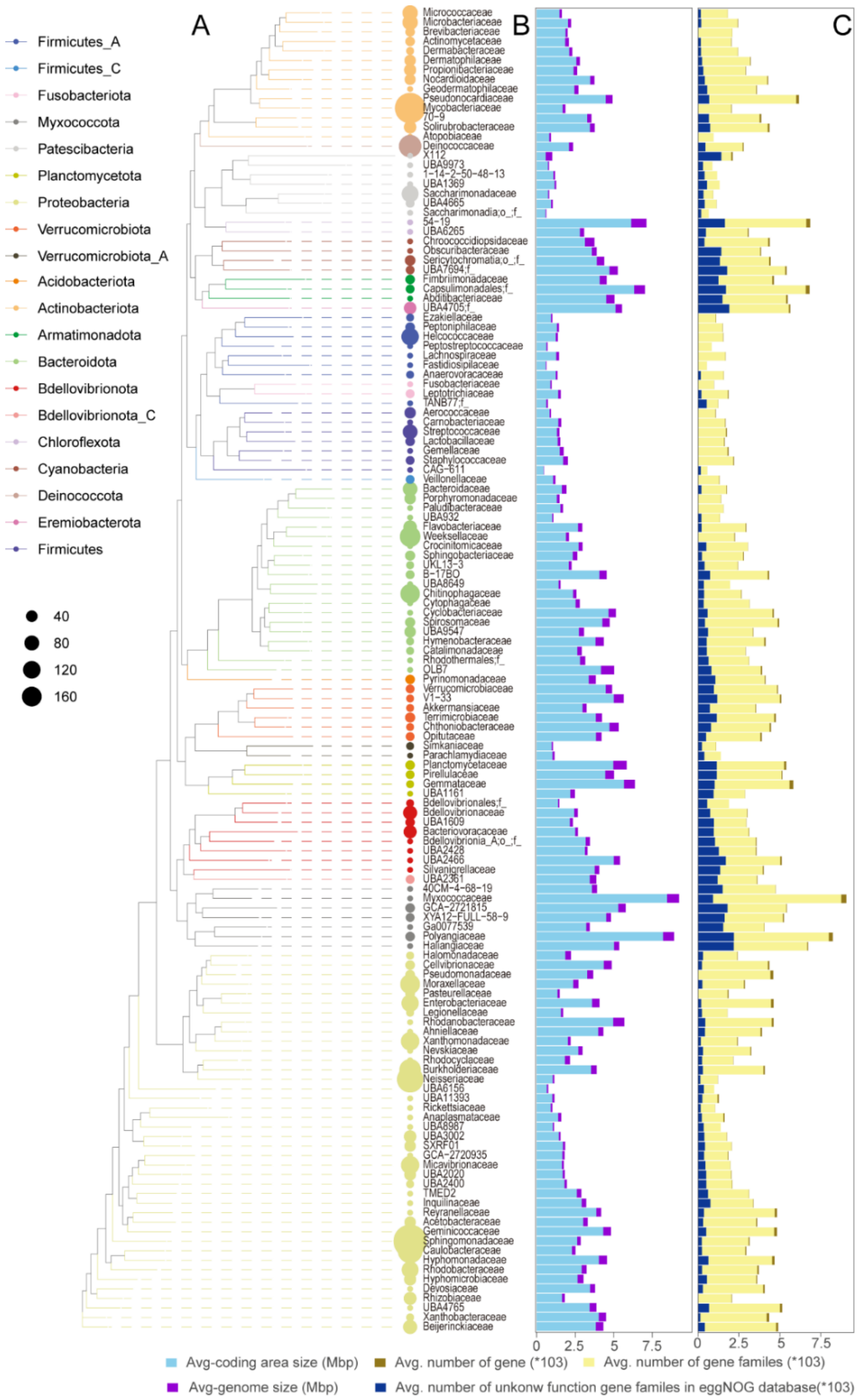

Figure S3. Diversity of composition and potential functional genes families of unclassified skin microbiota at the family level.

(A) The phylogenetic tree shows the phylogeny of 139 (family levels) bacterial clades of the unclassified MAGs. The outer circle of the clades shows the number of MAGs for each clade.

(B) The average genome size and coding density of all species in each branch are displayed.

(C) The histogram shows the average number of genes, the average number of gene families, and the number of unknown functional genes of all species in each branch.

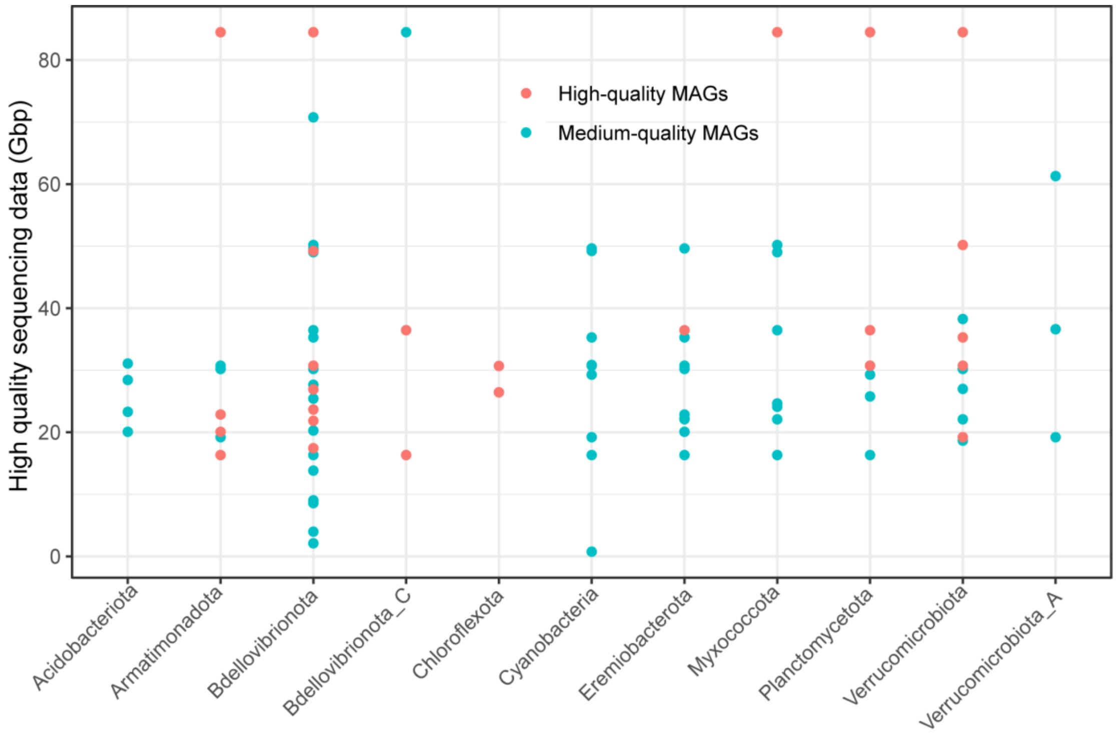

Figure S4. The "novel" discovered phylum in the skin was assembled from deep shot-gun metagenomic sequencing data samples.

The Y axis represents the sequencing data of each sample.

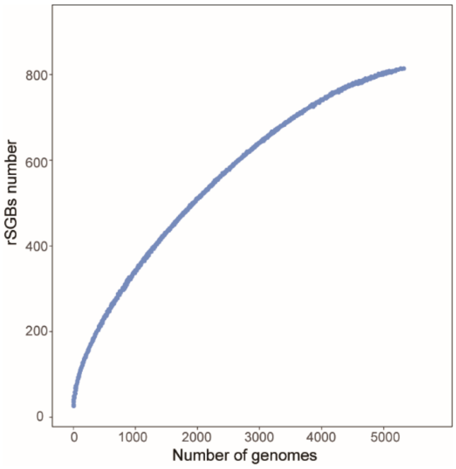

Figure S5. Rarefaction curves display the number of rSGBs obtained from the different number of genomes.

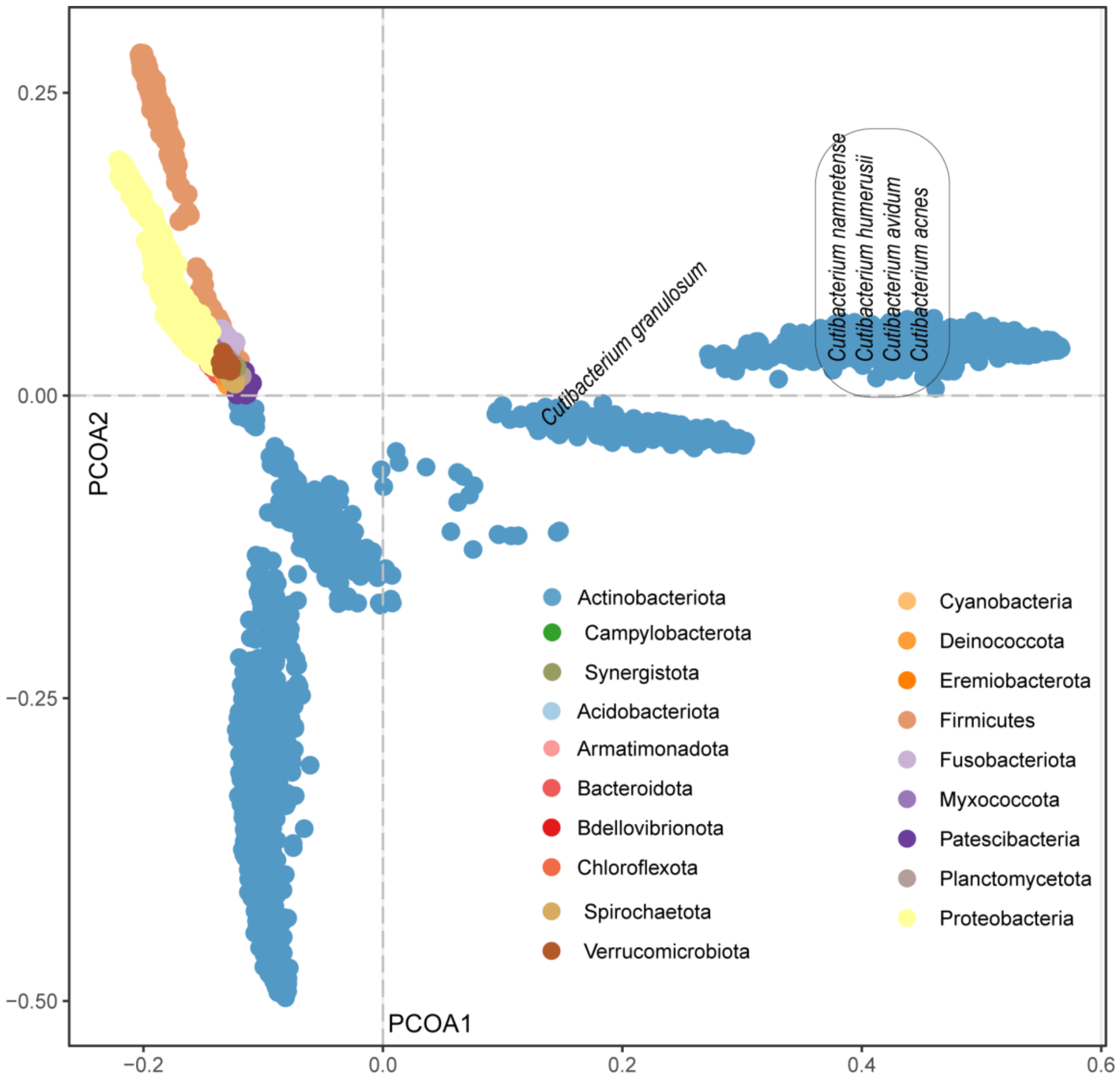

Figure S6. PCoA analysis of gene families shows a clear separation of functions among four different phyla.

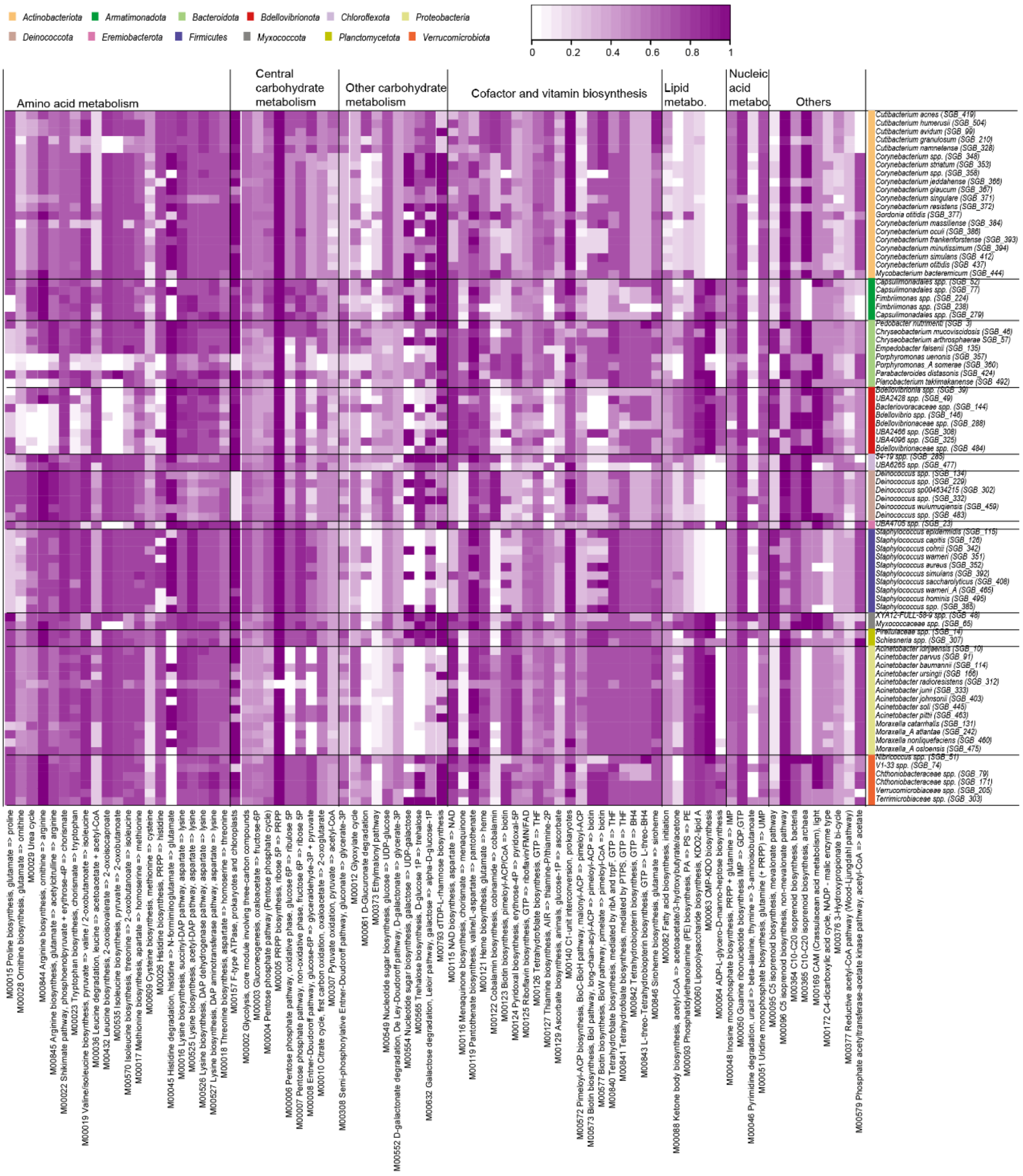

Figure S7. The potential core function module of skin bacteria.

The heatmap indicates the completeness ratio of lipid, carbohydrate, amino acid metabolism, and other pathways for skin bacteria. The color shows the completeness ratio of KEGG metabolic modules in NCPS; dark purple, genes with coding all enzymes involved in the module were present; light purple, partially present; white, none.

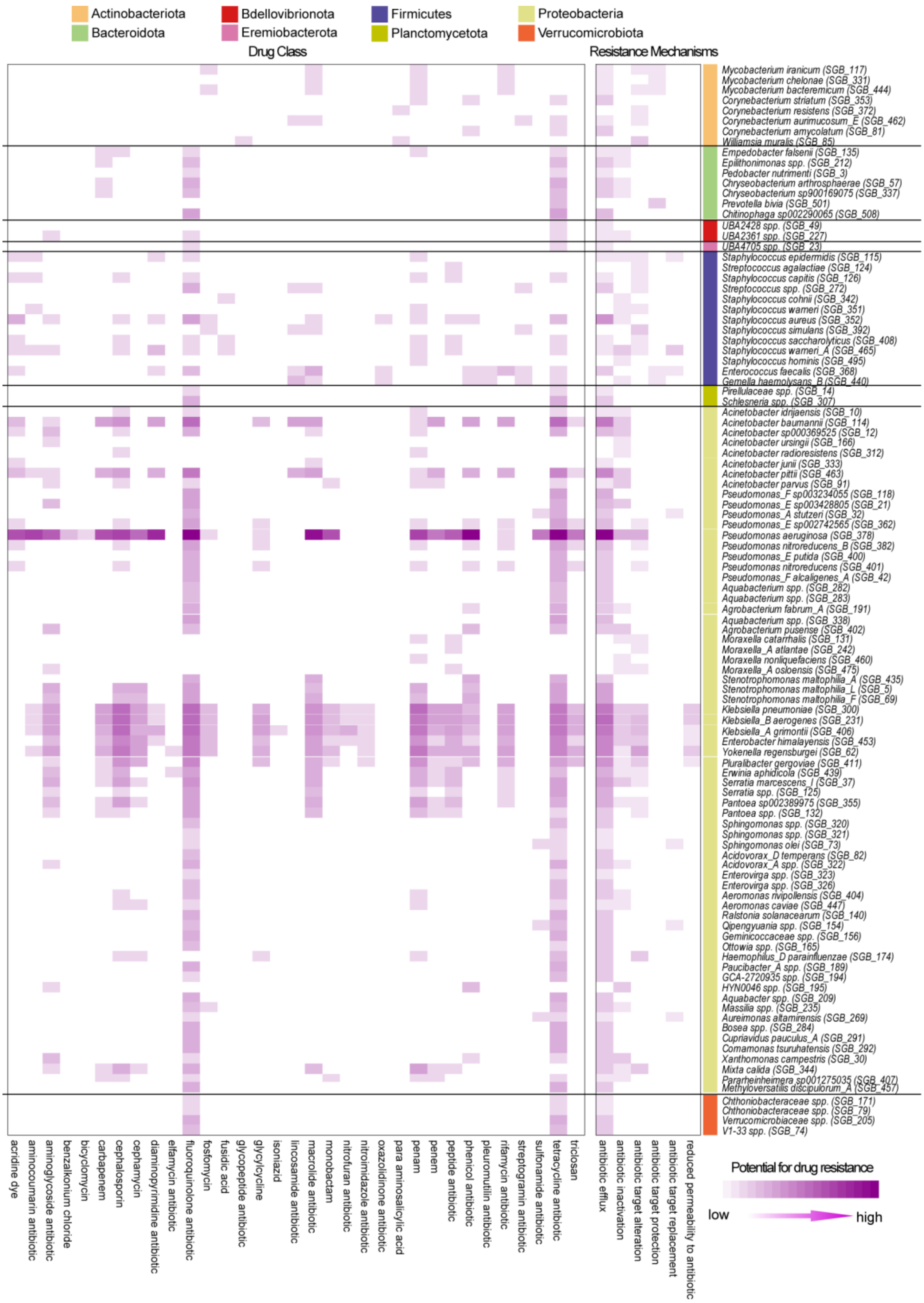

Figure S8. The difference in potential drug resistance of skin bacteria at different phylum levels. The color shows the number of resistance genes in NCPS. Darker colors reflect a larger number of genes.

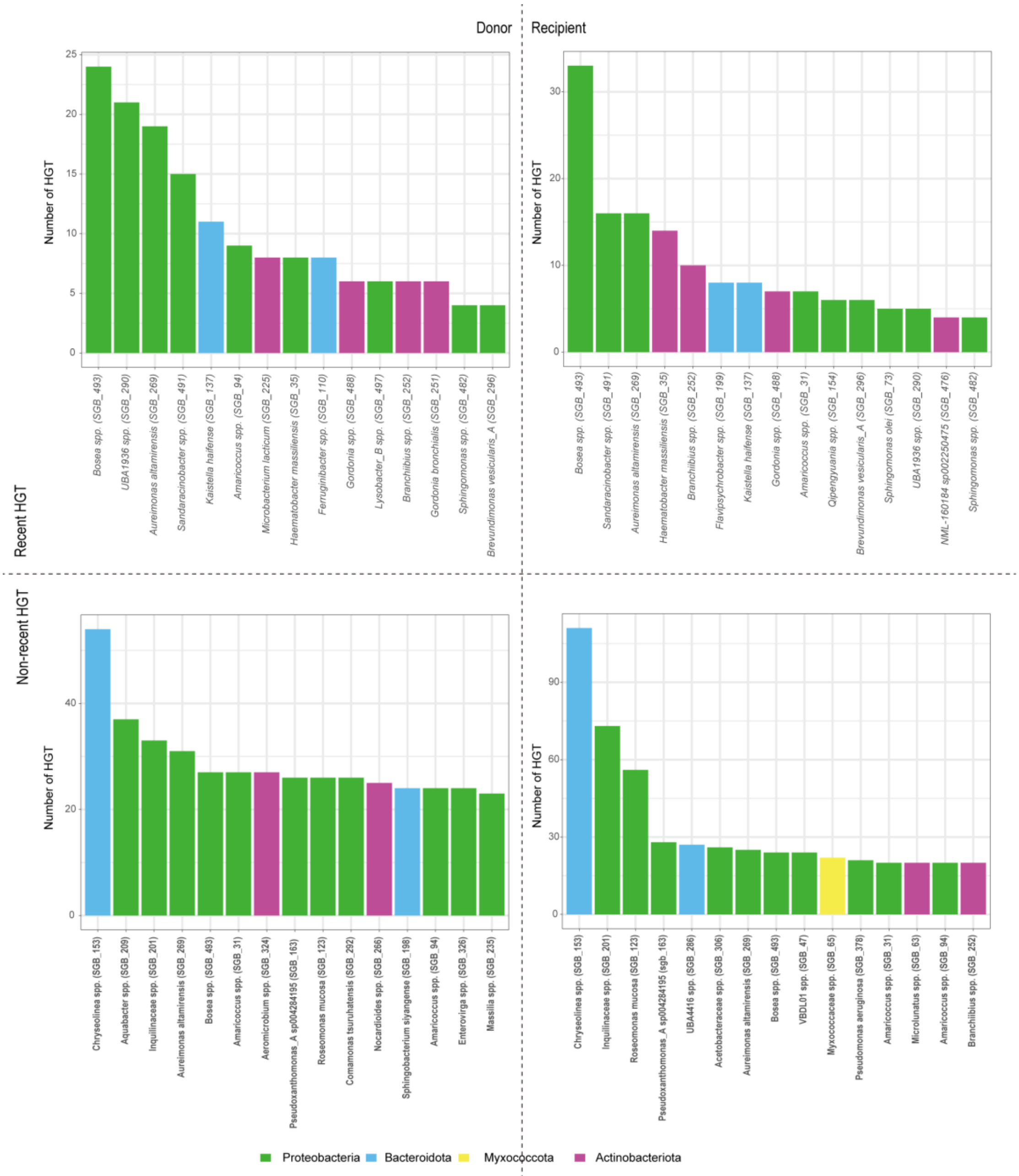

Figure S9. Donor and recipient of gene horizontal transfer at the species level.

The histogram shows the number of genes transferred horizontally, and the color represents the information of the phylum. Here are the top 15 with the highest number of genes transferred horizontally.

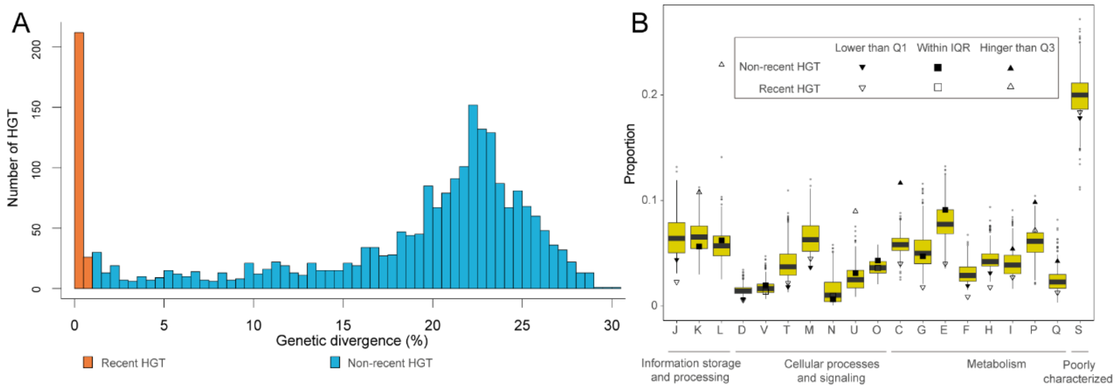

Figure S10. The effects of horizontal gene transfer on the potential function of skin bacteria. **(A)** Genetic divergence of MetaCHIP identified HGTs from the NCPS. **(B)** The relative proportion of COG functional categories in the NCPS and the predicted recent (genetic divergence ≤ 1%) and non-recent (genetic divergence > 1%) HGTs. The top and bottom of the box in the figure are composed of 25%-75% quartiles, and the thick line is the median. Q1, Q3, and IQR refer to 25%, 75%, and interquartile ranges respectively. The upper whisker refers to the largest observation less than or equal to upper Q3 + 1.5 × IQR, while the lower whisker refers to the smallest observation greater than or equal to Q1 − 1.5 × IQR. The letters on the x-axis indicate the COG category: J- translation, ribosomal structure and biogenesis, K-transcription, L- replication, recombination and repair, D- cell cycle control, cell division, and chromosome partitioning, V- defense mechanisms, T- signal transduction mechanisms, M- cell wall/membrane/envelope biogenesis, N- cell motility, U- intracellular trafficking, secretion, and vesicular transport, O- posttranslational modification, protein turnover, and chaperones, C-energy production and conversion, G- carbohydrate transport and metabolism, E- amino acid transport and metabolism, F- nucleotide transport and metabolism, H- coenzyme transport and metabolism, I- lipid metabolism, P- inorganic ion transport and metabolism, Q- secondary metabolites biosynthesis, transport, and catabolism, S- function unknown.

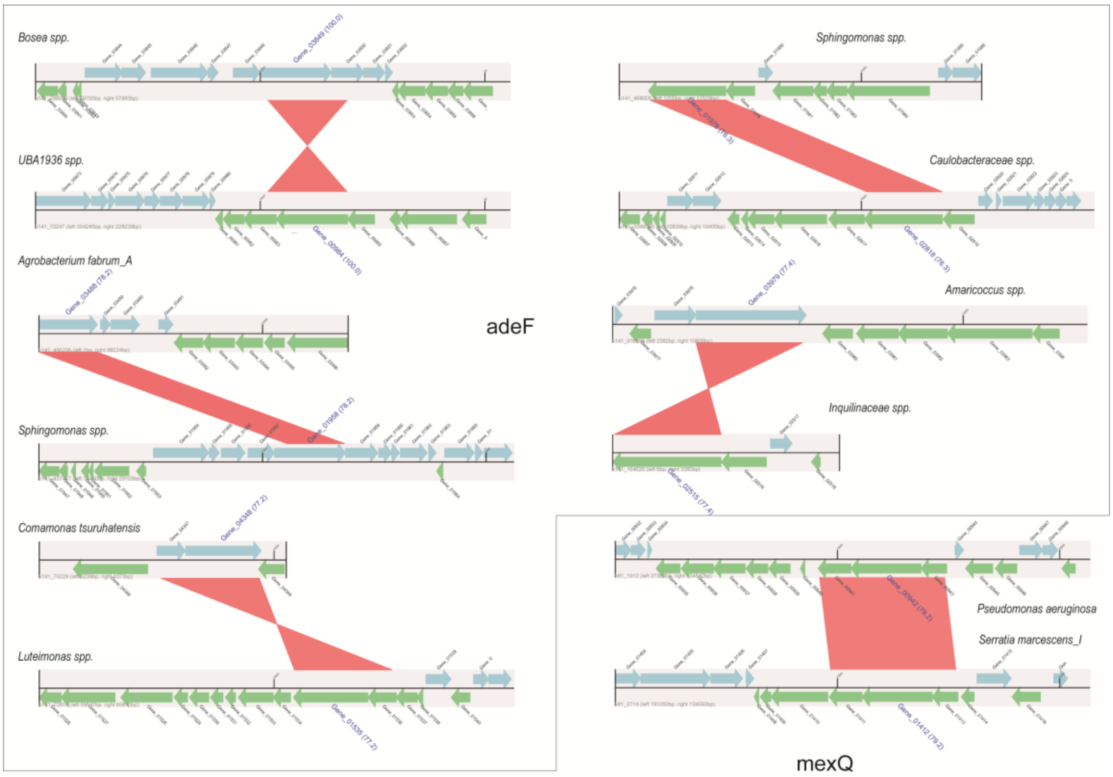

Figure S11. Drug-resistance genes of HGTs.

The genes encoded on the forward strands are shown in light blue, and the genes encoded on the reverse strands are shown in light green. The names of genes predicted to be HGT are highlighted in blue. The red bars display the similarity of matching regions between the contigs based on BLASTN results.

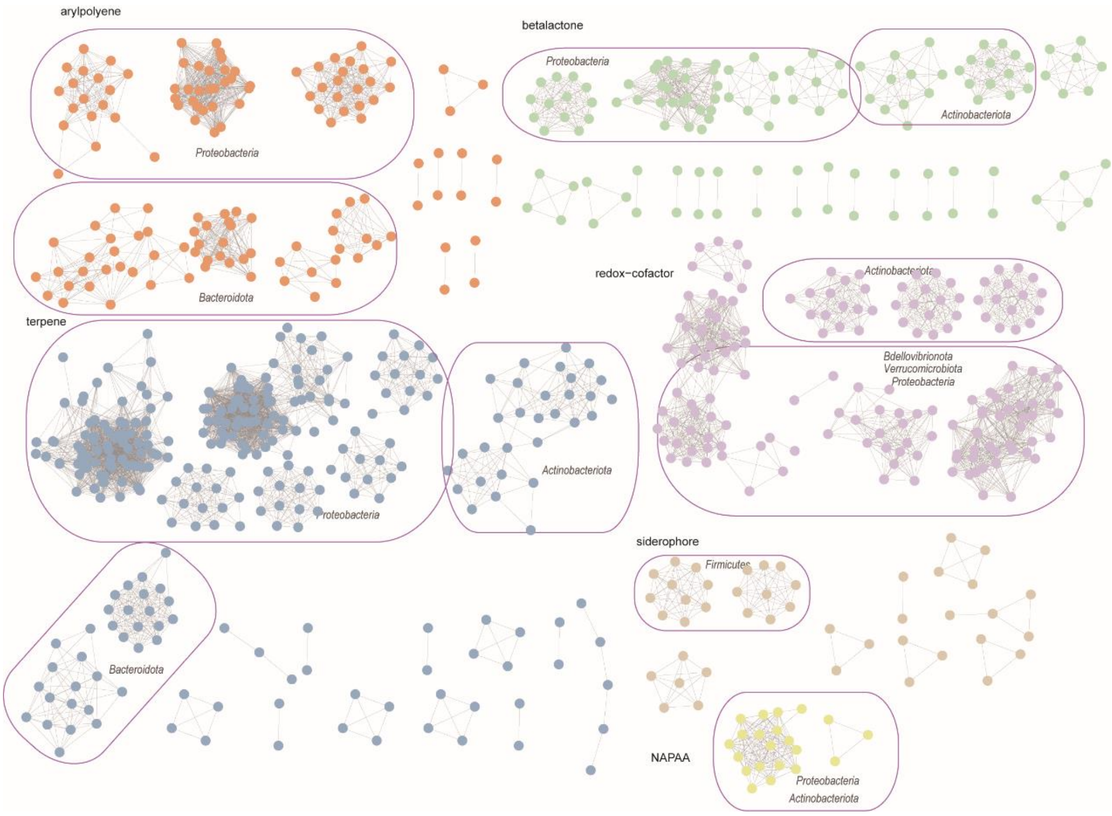

Figure S12. A network of biosynthetic gene clusters except for NRPS/PKS.

A network of biosynthetic gene clusters in which edges connect clusters that share genes. The line thickness increases with an increase in genetic similarity. The color represents the secondary metabolite corresponding to the biosynthetic cluster.

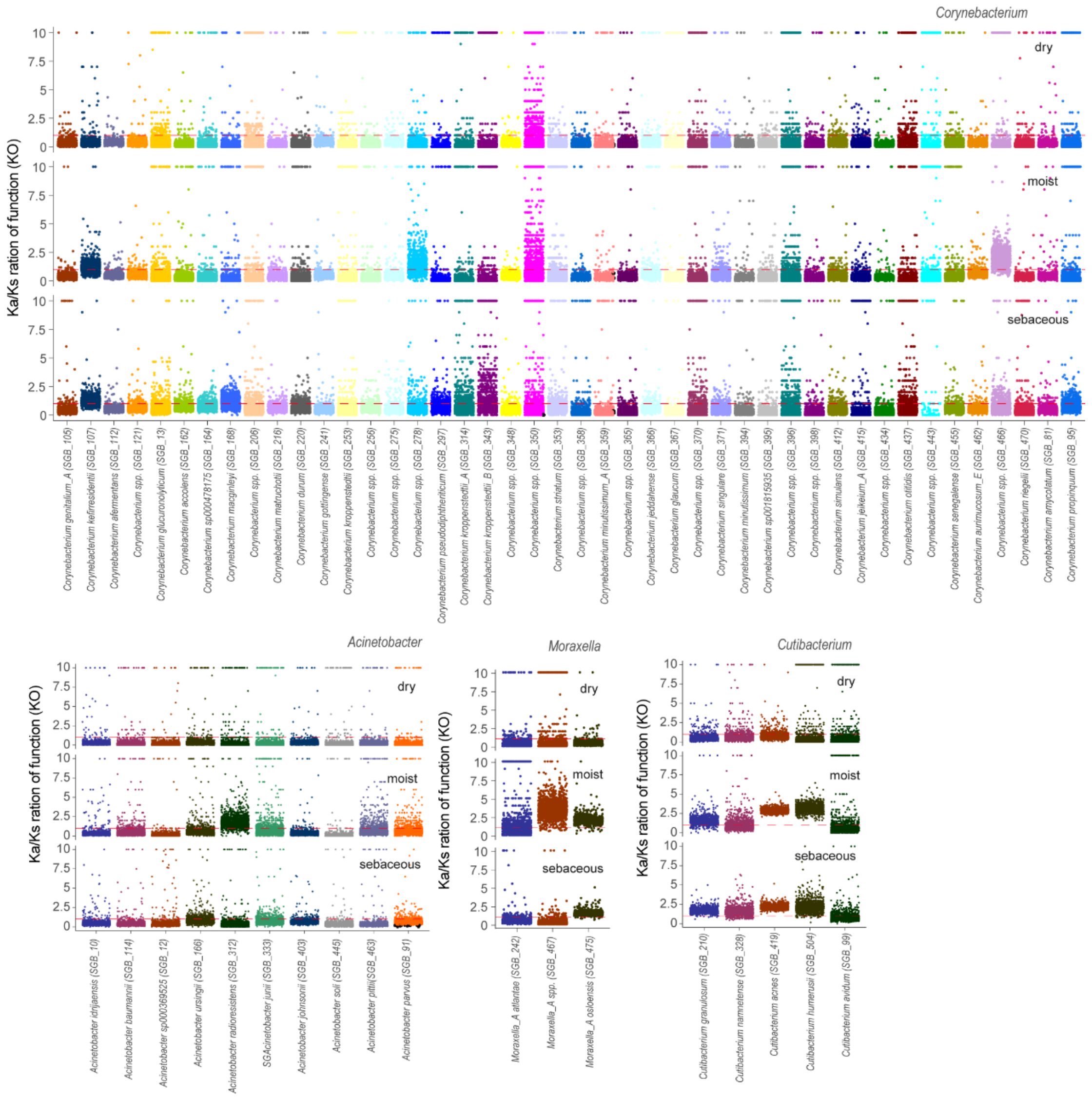

Figure S13. The Ka/Ks differences in potential functions between different species of *Corynebacterium, Acinetobacter, Moraxella,* and *Cutibacterium* in different skin environments.

Supplementary Materials Tables

Table S1. Sequencing statistics for 4D-SZ cohort skin metagenomic and publicly skin metagenomic datasets.

Table S2. MAGs statistics of the 5779 human skin microbial genomes.

Table S3. The average gene number, gene families number, and novel function number of MAGs at each order level and family level.

Table S4. General statistics of the 813 representative species within the UHSG catalog.

Table S5. Functional differences among different species of Cutibacterium.

Table S6. Detailed information on the skin microbial ARGs.

Table S7. Detailed information on the skin microbial HGTs.

Table S8. Detailed information on the skin microbial BGCs.
